# Development of Curcumin Encapsulated Liposomes in Chlorhexidine-Loaded Organogel Using Ternary Phase Systems for Treatment of Omphalitis in Infants

**DOI:** 10.1155/adpp/6828052

**Published:** 2025-01-30

**Authors:** Ibilola Mary Cardoso-Daodu, Margaret Okonawan Ilomuanya

**Affiliations:** Department of Pharmaceutics and Pharmaceutical Technology, Faculty of Pharmacy, University of Lagos, PMB 12003, Lagos, Nigeria

## Abstract

Infections in infants, after childbirth, remain a leading cause of neonatal morbidity and mortality, globally. A soaring percentage of these infections arise from bacterial colonization of the umbilicus. Current therapy for omphalitis includes the topical application of chlorhexidine on the umbilicus. Bacteria such as *Pseudomonas aeruginosa, Escherichia coli, and Staphylococcus aureus,* which are the key causative organisms of omphalitis, are resistant to chlorhexidine. In this study, curcumin-loaded liposomes were prepared using the “thin film hydration” method. Liposomes were characterized by particle size analysis, light microscopy, encapsulation efficiency, and flux. Stable organogels were formed via a high-speed homogenization method and stabilized by an emulsifier mix. They were evaluated for stability over a period by observing for phase separation. Four gels F1 (curcumin-loaded liposomes in chlorhexidine organogel), F2 (curcumin-loaded liposomes in organogel), F3 (chlorhexidine in organogel), and control (plain organogel) were prepared. Physicochemical properties of all gels were evaluated such as organoleptic tests, gel-to-sol transition, rheological studies, pH, skin irritancy, spreadability, accelerated stability, and antibacterial activity studies. Liposomes were spherical with an average size of 7 μm and an encapsulation efficiency of 97%. The *in vitro* release profile best fits the Higuchi mathematical model implying that curcumin release was by diffusion and dissolution mechanism. *In vitro* release was also higher at pH 5.5. F1 had the highest spreadability of 63 mm^2^g^−1^ and the lowest viscosity of 184,400 MPas at a shear rate of 10 rotations per minute with a pH of 6.5. Formulation F1 also displayed the highest antibacterial activity against all three bacteria. It can be concluded that the synergistic interaction between curcumin and chlorhexidine may be responsible for the significant antibacterial potency exhibited in formulation F1. Curcumin-loaded liposomes in chlorhexidine organogel (F1) can serve as a prototype for the development of an antibacterial topical formulation having intrinsic activity and enhanced potency to combat omphalitis.

## 1. Introduction

Infections in infants, after childbirth, remain a leading cause of neonatal morbidity and mortality, globally [1]. A soaring percentage of these infections arise from bacterial colonization of the umbilicus [2]. Umbilical cord healthcare practices vary depending on cultural practices in different geographical zones. The umbilicus proves to be an ideal microenvironment for bacterial growth and provides immediate access to the bloodstream of an infant. Bacterial colonization of the umbilical cord results in omphalitis [3]. Omphalitis can rapidly progress to a systemic infection, leading to sepsis and death. Omphalitis has an estimated mortality rate of 15% in neonates globally, while in Nigeria, morbidity is 21.8%. This is significantly higher than the global mortality rate [4].

**Figure 1:**
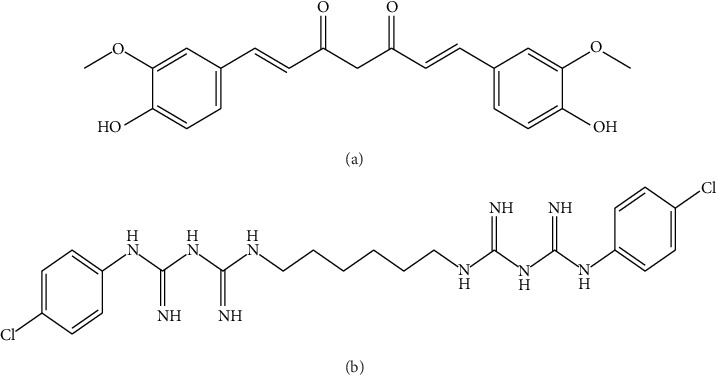
(a and b) Chemical structure of diferuloylmethane (curcumin) and 1, 1′-hexamethylenebis(5-[p-chlorophenyl]biguanide)chlorhexidine, both have antibacterial properties.

Current therapy for omphalitis includes the topical application of chlorhexidine on the umbilicus. Chlorhexidine is a biguanide compound composed of a cationic biguanide consisting of two 4-chlorophenyl rings and two biguanide groups joined by a central hexamethylene chain [5]. Chlorhexidine is a positively charged molecule. It reacts with negatively charged phosphate groups present on bacteria cell surfaces leading to the destruction of the integrity of the bacterial cell surface via leakage of intracellular material [6]. This causes precipitation of cytoplasmic components and ultimately bacteria cell lysis. Chlorhexidine is also established as a broad-spectrum antibacterial agent with antibacterial activity against both gram-positive and gram-negative bacteria [7]. Recently, bacteria such as *Pseudomonas aeruginosa*, *Escherichia coli*, and *Staphylococcus aureus* which are causative micro-organisms in omphalitis have developed some resistance against chlorhexidine, hence, its coformulation with curcumin to achieve a synergistic effect potent enough to eliminate bacteria [8]. The chemical structures of curcumin and chlorhexidine are illustrated in Figures 1(a) and 1(b).

Curcumin is an active compound found in the rhizome of *Curcuma longa* (Zingiberaceae). It is a member of the ginger family. Curcumin exhibits antibacterial activity against a range of bacterial species [9]. Curcumin blocks bacteria growth, and this occurs, owing to its structural characteristics via a variety of mechanisms [10]. Curcumin can inhibit bacterial virulence factors, inhibit bacterial biofilm formation, and prevent bacterial adhesion to host receptors through the bacterial quorum sensing regulation system. Quorum sensing is a process through which chemical signaling molecules call autoinducers to regulate bacterial gene expression. Curcumin also induces oxidative stress leading to bacterial death owing to the presence of epoxide moieties [11]. The coformulation of curcumin and chlorhexidine as a single formulation promises a synergistic effect unsurmountable by bacterial organisms. This ensures the complete elimination of the polymicrobes at the colonized umbilicus. Curcumin is a lipophilic drug molecule, and this characteristic must be considered during the development of a formulation for drug delivery. Curcumin can be conveniently encapsulated in liposomes to improve its water solubility as a drug molecule.

**Figure 2:**
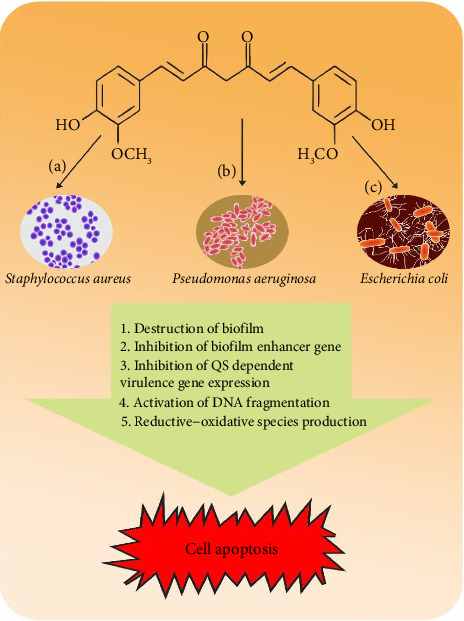
Schematic illustration of the mechanism of action of curcumin against *Pseudomonas aeruginosa, Escherichia coli, and Staphylococcus aureus.*

The schematic illustration of the mechanism of action of curcumin against the causative organisms of omphalitis is shown in Figure 2.

Liposomes are nanosized or microsized vesicles comprising a phospholipid bilayer that enclose an aqueous core. The inner core encapsulates the hydrophilic drugs and the hydrophobic domain is responsible for entrapping poorly soluble lipophilic agents [12]. Liposomes are suspensions that easily run off the skin and, therefore, may not be easily applied topically due to the limited contact time between the formulation and the skin [13]. An ideal solution would be to incorporate the curcumin-loaded liposomes into a viscous triphasic polymer such as an organogel to increase dermal contact time. The organogel polymer forms a film around the lipid bilayer membrane of the liposomes increasing its rigidity and also enhancing its stability [14].

Organogels are a class of gels composed of a liquid organic phase within a three-dimensional crosslinked network. Organogels can house loaded bioactive molecules within their network for absorption into the skin. Ternary phase diagrams represent ternary systems such as organogels [15]. These phase diagrams reflect the phase behavior of three component mixtures. Organogels are composed of an organic phase, an aqueous phase, and a surfactant or surfactant mix. A ternary phase diagram shows possible phases and their equilibrium according to the composition of the organogel mixture at constant temperature and pressure [16].

The purpose of this study is to formulate *Elaeis guineensis* (oil extract)–based organogel as a carrier for the topical delivery of chlorhexidine and curcumin-loaded liposomes in the treatment of omphalitis. This study utilizes the raw materials, *Elaeis guineensis,* and curcumin to develop a pharmaceutical formulation. This is a novel concept in the field of drug delivery for infants. Current treatment of omphalitis involves the use of a cocktail of oral and intravenous antibiotics, too expensive for the average Nigerian who lives on less than a dollar a day. The potency of chlorhexidine has been reduced due to the development of bacterial resistance*. Elaeis guineensis* oil extract also has well-established antimicrobial properties. It will serve as an ideal vehicle for the delivery of curcumin-loaded liposomes and chlorhexidine. Successful development of this novel formulation will lead to an improved and affordable prognosis of omphalitis in infants [17].

## 2. Materials and Methods

### 2.1. Materials

Materials used in this experiment are as follows: Tween 80 and Span 20 (Merck NJ, United States of America), distilled water (Central Research Lab, CMUL, Idi-Araba, Lagos), chlorhexidine (*Elaeis guineensis* oil [Maubold Essensce, Lagos, Nigeria]), curcumin and phosphatidylcholine (Sigma-Aldrich Co., St. Louis, Missouri, United States of America), methanol (Merck, Darmstadt, Germany), phosphate buffer (Loba Chemie, Colaba, Mumbai, India), 1% cremophor (RH40) (Macklin Biochemical, Shanghai, China), Carbopol Ultrez (Surfachem, United Kingdom), distilled water, chlorhexidine (Sigma-Aldrich Co., St. Louis, Missouri, United States of America), triethanolamine (Merck, New Jersey, USA), Dettol (Reckitt Benckiser), normal saline (Unidex, Lagos, Nigeria), Mueller–Hinton agar (HiMedia Laboratories), and methylparaben and propylparaben (Sigma-Aldrich Co., St. Louis, Missouri, United States of America).

### 2.2. Method

#### 2.2.1. Preparation of Curcumin-Loaded Liposomes

**Figure 3:**
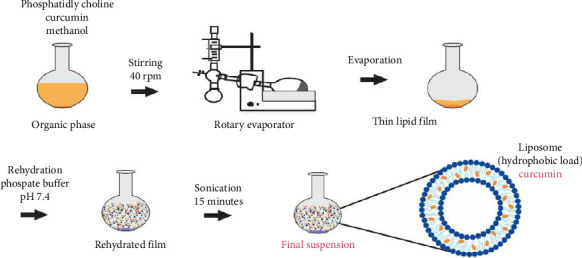
Schematic diagram of the preparation of curcumin-loaded liposomes using the thin lipid film hydration method.

Phosphatidylcholine (10 mg) and curcumin (4.35 mg) were weighed and dissolved in methanol (25 mL) in a round-bottomed flask. The mixture was attached to a rotary evaporator for 30 min. A thin orange lipid film was observed at the bottom of the flask [14]. The film was then rehydrated using phosphate buffer (pH 7.0). The rehydrated mixture was sonicated for 15 min and then vortexed for 5 min. The liposomes were stored in the refrigerator at 4°C. An illustration of the process of preparation of curcumin-loaded liposomes is shown in Figure 3 [18, 19].

#### 2.2.2. Characterization of Liposomes

##### 2.2.2.1. Light Microscopy

Light microscopy was employed in the characterization of the morphology of the prepared liposome formulation. The lipid suspension was mounted on a slide and a clear cover slip was placed on top of it, before viewing under the microscope (XSZ-107BN-A Olympus microscope, OHAUS NJ, United States of America) at varying magnifications [20].

##### 2.2.2.2. Encapsulation Efficiency (Centrifugation)

Curcumin-loaded liposomes (5 mL) were transferred into a centrifuge tube and allowed to centrifuge for 15 min at 400 rotations per minute. The supernatant separated from the curcumin-loaded liposome which had settled at the bottom. The curcumin-loaded liposome suspension was taken for UV analysis at *λ*_max_ 425 nm. Encapsulation efficacy was calculated using equation (1). The experiment was performed in triplicates [21].

(1) $$\begin{array}{c} \text{Encapsulation efficiency}=\left(\frac{\text{total concentration of curcumin}-\text{concentration of free curcumin}}{\text{total concentration of curcumin}}\right)x100. \end{array}$$

##### 2.2.2.3. *In Vitro* Curcumin-Loaded Liposome Release Studies

A modified Franz cell apparatus was utilized for the analysis of the *in vitro* drug release of curcumin. Cremophor (pH 6.0–8.0) RH40 was added to a phosphate buffer of pH 7.0 to give the diffusion fluid. The receiver compartment was filled with the already prepared diffusion fluid. A Millipore membrane filter was soaked in the diffusion fluid for 1 h. The Millipore filter membrane was used to cover the lower part of the donor compartment and to secure it with a ring. The modified Franz diffusion apparatus was fixed on a magnetic stirrer and heated to 37°C. The curcumin-loaded liposomal formulation (1 mL) was introduced into the donor compartment. Precisely, 1 mL of the sample was drawn from the diffusion fluid and replaced with fresh diffusion fluid at intervals of 5, 10, 15, 30, 60, 120, 180, 240, 300, 360, and 420 min. All samples from the various time points were taken for UV analysis at a wavelength of *λ*_max_ 425 nm. This experiment was performed in triplicates [22].

#### 2.2.3. Preparation of Organogels

Tween 80 and Span 20 were blended in varying amounts to obtain a unique emulsifier mix according to Table 1. The emulsifier mix was added to different amounts of *Elaeis guineensis* at 60°C while stirring with a magnetic stirrer at 500 rotations per minute for 30 min. Distilled water was then added in drops until a triphasic gel system was formed. Ternary plots were constructed using tri-plot software to give the area of gelation at different ratios of cosurfactants, oil, and aqueous phase. To determine the most stable organogel and the best emulsifier mix, the organogels A1–C3 were assessed for gel-to-sol transition temperatures after they were freshly prepared and 3 days after storage. Samples of each organogel were collected at different temperature points and subjected to the inverted test-tube method. Organogels were also stored at 25°C and 4°C after which they were observed visually for any change such as phase separation [15].

#### 2.2.4. Preparation of Loaded Organogels (F1–F3 and Control)

The ideal proportion of emulsifier mix (1:1 of Span 20 and Tween 80), water, and oil, were detected using the ternary plot. According to Table 2, the different amounts of curcumin-loaded liposomes and chlorhexidine were added to the aqueous phase (water). A specific amount of emulsifier mix was added to the specific amount of *Elaeis guineensis* at 60°C while stirring with the aid of a magnetic stirrer at 500 rotations per minute for 30 min. The required amount of distilled water was then added in drops until the organogel system was formed [15].

#### 2.2.5. Characterization of Organogel Formulations

##### 2.2.5.1. Organoleptic Test

The color, texture, odor, homogeneity, and consistency of the F1–F3 and control formulations were evaluated and recorded [23].

###### 2.2.5.1.1. Rheological Properties

The viscosity of F1–F3 and control was obtained at room temperature (37°C) at 10–100 rpm using a spindles cone and plate viscometer (NDJ-1F Brookfield Rotational Viscometer, Brookfield Engineering Laboratories, China) [24].

##### 2.2.5.2. pH

The organogel formulations F1–F3 and control were evaluated for their pH using a pH meter (SevenCompact S220 Basic, Mettler-Toledo, Switzerland). An electrode was immersed in the formulation for 45 s and the pH was read [25].

##### 2.2.5.3. Skin Irritancy

Animal ethics approval was obtained with approval number: CMUL/ACUREC/04/24/1473. The organogel formulations F1–F3 and control (0.3 g) were applied to the bare skin (3 cm^2^) on the dorsal back of a 3–4 weeks' infant Wister rat and observed for 4 h for signs of erythema, edema, or rash [24].

##### 2.2.5.4. Spreadability

The organogel formulations F1–F3 and control were assessed for spreadability. Precisely, 0.5 g of each loaded organogel formulation was applied in between two glass slides and a 100 g mass was placed on both sides for 1 minute to compress the gel formulation and obtain uniform thickness. Extra gel at the edges of the slide was wiped off. Another 50 g mass *M* was attached to the upper slide. The time required to move the slide across a distance of 20 cm was taken as the level of spreadability [26].

The following equation is used for calculating spreadability:

(2) $$\begin{array}{c} S=M\frac{L}{T}, \end{array}$$

 where *M* is the weight tied to the upper slide, *L* is the length of the glass slide, and *T* is the time taken to separate the slides.

##### 2.2.5.5. Stability Studies

• Accelerated stability
The stability test was carried out for organogel formulations F1–F3 and control, using the thermal-cycling method [27].
  • Phase I
    Heating and cooling cycle between 4°C and 37°C with storage at each temperature for a minimum of 48 h. Samples were centrifuged at 3500 RPM for 15 min to observe phase separation or drug precipitation [27].
  • Phase II
    Organogels are subjected to three cycles of alternate temperatures −4°C and 25°C for a minimum of 48 h. Samples are then centrifuged at 3500 RPM for 15 min [27].
• Long-term stability
Organogels are kept at (atmospheric temperature) 24°C, (fridge) 4°C, and (body temperature) 37°C at 60% RH. The stability of the organogels is observed on Days 1, 7 30, 60, 90, and 120 [28].
• Antibacterial activity testing (well diffusion method)
Animal ethics approval was obtained with approval number: CMUL/ACUREC/04/24/1473. *Pseudomonas* aeruginosa was isolated on cetrimide agar, *Escherichia coli* was isolated on eosin methylene blue agar, while the *Staphylococcus aureus* was isolated on mannitol salt agar. All the agars were selective media that suppressed the unwanted organisms and allowed the bacteria of interest to grow. All three bacteria were then subcultured onto Mueller–Hinton agar to remove the effects of suppressive chemical agents in primary isolation media and then incubated at 37°C for 24 h. Antibacterial activity testing was conducted using a well diffusion method. This was carried out on Mueller–Hinton agar plates. Cultured bacteria samples were evenly streaked on Mueller–Hinton agar plates. Then wells were bored into the plates and filled with the organogel formulations F1–F3 and control, respectively. Plates were then incubated at 37°C for a day. Zones of inhibition were observed and the diameters of the zones of inhibition were measured [29–32].

##### 2.2.5.6. Statistical Studies

Experiments were performed independently in triplicates, and the data provided are the mean ± standard deviation. One-way ANOVA was applied in the statistical analysis of data, where *p* < 0.05 was considered statistically significant [33].

## 3. Results

### 3.1. Light Microscopy and *In Vitro* Release of Curcumin Using Phosphate Buffer Media pH 7.0 and pH 5.5

**Figure 4:**
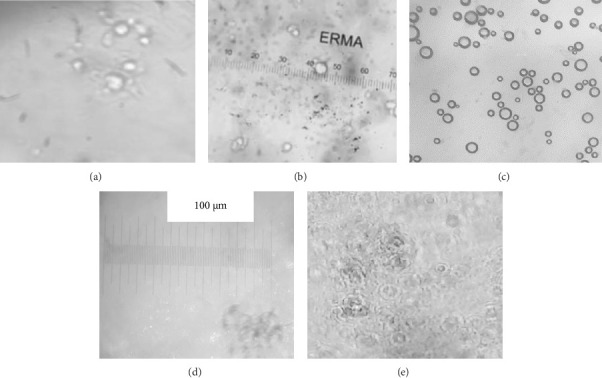
Light microscopy images of curcumin-loaded liposomes in phosphate buffer pH 7.0 at different magnifications. Liposomes at x 40 magnification (a and b) x 400 magnification (c) x 10 magnification (d) and x 30 magnification (e).

The size and morphology of the liposomes were examined using a light microscope. Liposomes were spherical in shape with an average of about 7 μm in size. The encapsulation efficiency of the curcumin-loaded liposomes was 97%; this shows that the liposomes were stable and encapsulated the drug curcumin efficiently. Figures 4(a), 4(b), 4(c), 4(d), and 4(e) show the microscopic images of the curcumin-loaded liposome suspension at varying magnifications.

The encapsulation efficiency is influenced by the concentration of phospholipids, the sonication time, and the technique used in the preparation of liposomes. These factors primarily influence the surface charge, phospholipid molecule alignment, and bilayer membrane fluidity, which in turn determine the shape, size, and stability of the liposome. Sonication in particular reduces liposomes' size by creating cavitation which is fostered by oscillating bubbles that produce a shear field via sound energy.

**Figure 5:**
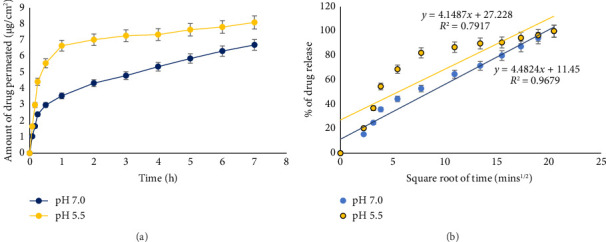
Release profile of curcumin from loaded liposomes at pH 7.0 and pH 5.5 at different time intervals and (a) Higuchi mathematical model's release profile of curcumin from loaded liposomes at pH 7.0 and pH 5.5 at different time intervals (b).

Liposomes with large diameters collide with this shear field and morph into micelle-like appendages that subdivide to give smaller liposomes. The *in vitro* analysis of the release of curcumin from the liposomes was assessed at pH 7.0 and 5.5. It was observed that there was a rapid release of curcumin at both pHs for the first few minutes. Subsequently, the release followed a slower pattern. The total release of curcumin occurred throughout 7 h. The release behavior of curcumin from liposomes can be described as a sustained release pattern. There was a higher release of curcumin at a pH of 5.5 compared to 7.0 as seen in Figures 5(a) and 5(b), which show the Higuchi release plot for the curcumin-loaded liposomes.

### 3.2. Construction of Ternary Phase Diagrams A, B, and C

Ternary phase diagrams were plotted using three different ratios (1:1, 1:2, and 1:3) of cosurfactants (tween and span). It was clear from the results of the stability test that of all three ratios, ratio 1:1 produced the most stable organogel (A1), which was selected as the optimal.

**Figure 6:**
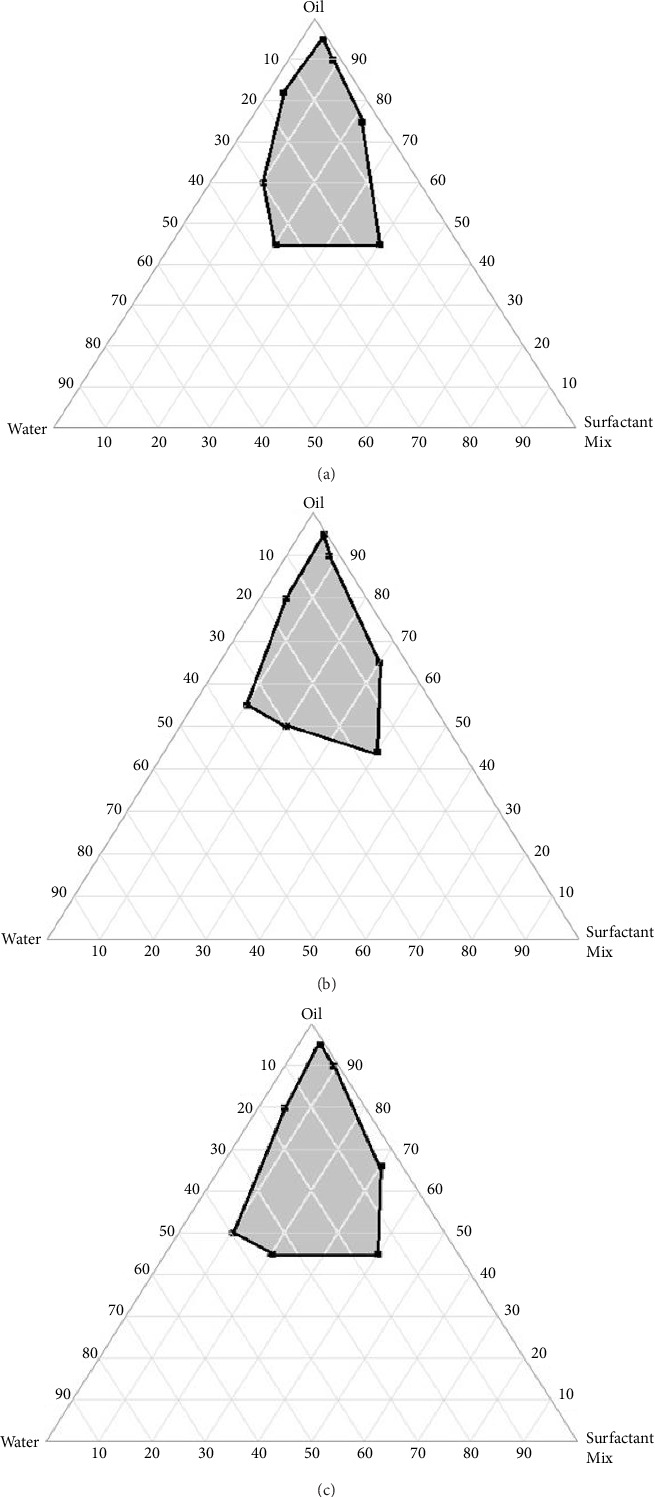
Pseudoternary phase diagram of organogel formulations made up of *Elias guineensis,* tween and span (1:1), and distilled water (a), *Elias guineensis*, tween and span (1:2), and distilled water (b), and *Elias guineensis*, tween and span, (1:3) and distilled water (c).

Organogel formulation for further studies: The ternary phase diagrams for the different ratios (1:1, 1:2, and 1:3) of the emulsifier mix are shown in Figures 6(a), 6(b), and 6(c). A1 had a high gel-to-sol temperature and remained stable in all storage conditions it was subjected to, at different time intervals.

### 3.3. Organoleptic Tests

**Figure 7:**
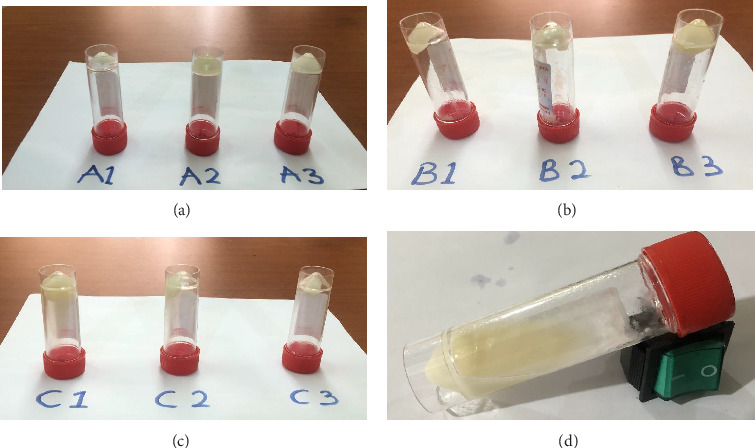
Side view photographs of prepared organogels with code names A1–A3 (a), B1–B3 (b), C1–C3 (c), and a phase-separated organogel (d) at room temperature (24°C).

All organogel formulations had a characteristic antiseptic odor due to the presence of chlorhexidine. The texture was smooth and the gel washed off easily using soap and water sequel to skin application. No sign of irritation, edema, or erythema was observed up until 24 h after the application of the gels.  Table 3 shows the appearance of all organogels stored at room temperature (24°C) and in the refrigerator (4°C). Figures 7(a), 7(b), 7(c), and 7(d) show a photographic representation of the organogels at room temperature when the organogels are inverted. Organogels A1, A3, B1, and C3 did not run over.

Based on the stability test and gel-to-sol assessment, Organogel A1 was the most stable and was selected for further formulations, that is, it was the triphasic drug delivery vehicle for curcumin-loaded liposomes and chlorhexidine.

### 3.4. The pH, Viscosity, and Spreadability of Liposome-In-Organogel Formulations (F1, F2, and F3 and Control)

**Figure 8:**
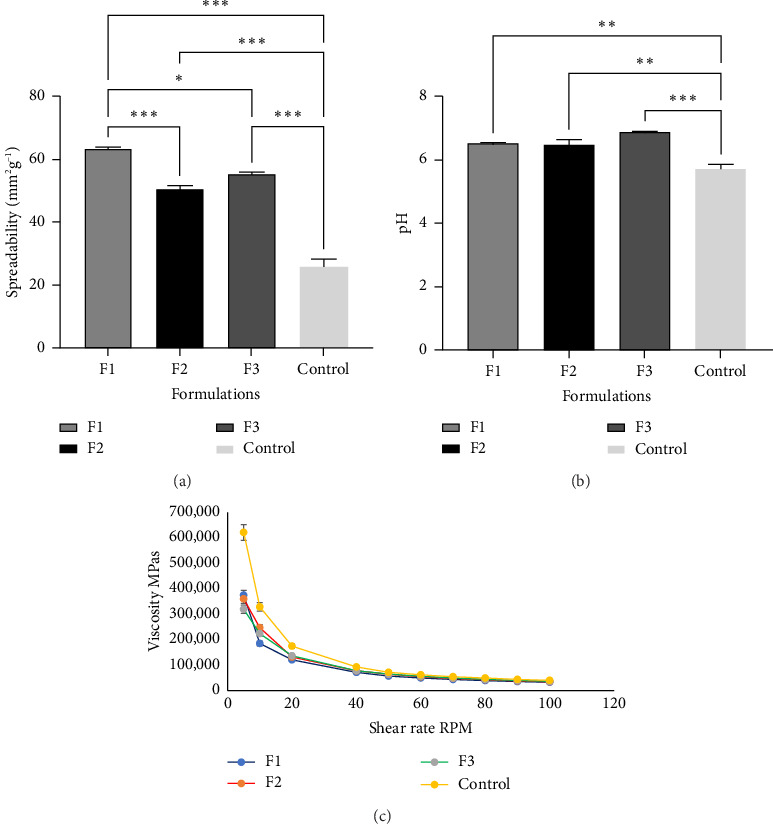
The spreadability (a) potential of hydrogen (b) and viscosity (c) of Formulations F1, F2, and F3 and control.

The pH of the gel formulations is vital as all formulations are intended for dermal application. The pH also has a role to play in the biocompatibility and safety of the gel concerning topical application. As seen in Figure 8(b) the pH of the organogels was within the range of 5.95–6.85, which is not far from the pH of the skin of an infant or newborn which is 5.5–6.5. The skin irritancy test was carried out by applying liposomes-in-organogel on the infant Wistar pups and checked at time intervals for 1 h. No edema or erythema was observed. Figures 8(a) and 8(c) show that the viscosity and spreadability of the organogels varied, and this was largely dependent on the composition of each formulation. The control had the highest viscosity, while formulations containing the curcumin-loaded liposomes and chlorhexidine had lower viscosities. The trend of the viscosity graph showed that as the shear rate increased, viscosity decreased, giving evidence that the topical formulations will spread with ease on dermal application by palm massage. The spreadability of the formulations is a measure of uniformity of the spread at the site of action on the skin. The least spreadable formulation was the control formulation followed by Formulation F2, while F1 had the highest spreadability. Formulations F1–F3 and control remained stable through the accelerated and long-term stability studies.

### 3.5. The Antimicrobial Activity of Formulations F1, F2, and F3 and Control Against *Staphylococcus aureus, Pseudomonas* aeruginosa, and *Escherichia coli* Was Assessed Using the Well Diffusion Method

**Figure 9:**
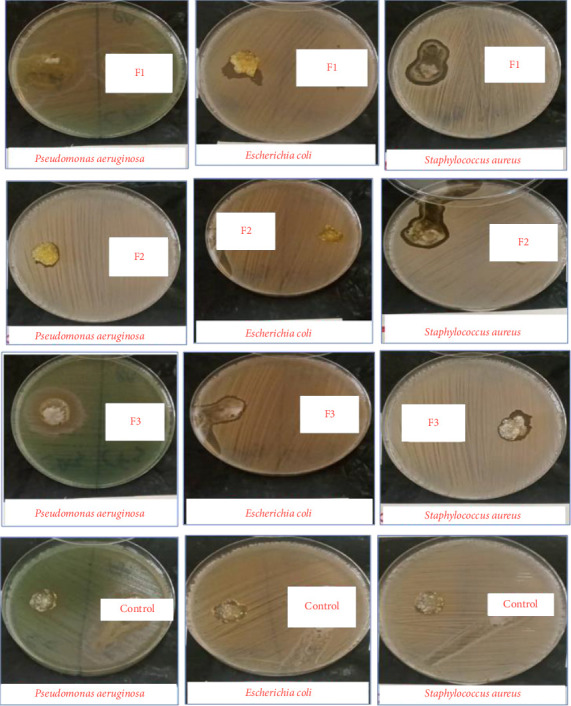
*In vitro* antibacterial activity of Formulations F1–F3 and control against *Staphylococcus aureus, Pseudomonas aeruginosa,* and *Escherichia coli*.

From the pictorial images in Figure 9 and data in Table 4, it appears that there was antibacterial activity against all three bacteria for Formulations F1 and F3, while for Formulation F2, there was no antibacterial activity against *Escherichia coli* and *Staphylococcus aureus*. The control formulation, however, did not show any antibacterial activity.

## 4. Discussion

Yearly, 4 million infant deaths occur in developing countries. Unfortunately, infections account for almost 40% of these deaths. Bacterial colonization of the umbilical cord leads to infection, sepsis, and death. Based on the existing literature, curcumin has been proven to possess intrinsic antibacterial properties [34]. A plethora of pharmacological pathways are responsible for curcumins' bactericidal properties. Curcumin can act by destroying the integrity of the bacterial cell membrane, preventing DNA replication, alteration in plasmid gene expression, and subsequently bacteria motility retardation. For purposes of this study, curcumin was loaded into liposomes to overcome some of its pharmacokinetic challenges such as short half-life, hydrophobicity, rapid metabolism, and low bioavailability [28].

In this study, the loading efficiency of the curcumin-loaded liposomes was 97%, implying that curcumin was efficiently loaded into the liposomes via passive loading and that the liposomes were stable and had enough lipophilic domain to house the curcumin molecules. The average size of the loaded liposomes was 7 μm, and the liposomes were spherical when observed using light microscopy. These characteristics as well as the morphology of the liposomes significantly influenced liposomal function especially in regards to permeability through the *stratum corneum* [35]. Liposomes can penetrate the stratum corneum where the liposomal membrane is dissolved facilitating a burst release effect. Liposomes release drugs via diffusion and dissolution mechanisms. This implies that after disruption of the liposome membrane which ushers a burst release effect, curcumin molecules automatically move from an area of higher concentration (within the liposome) to an area of lower concentration (stratum corneum) and dissolve within the stratum corneum.

Kinetic modeling of curcumin release via mathematical models is vital because understanding the model and release pattern aids optimization of drug release kinetics, achieving a desirable therapeutic activity. Deploying mathematical models to fit data generated from the *in vitro* release study in this investigation helps with understanding the release process of curcumin from the inner part of the liposomes [36]. *In vitro* analysis showed a higher release profile of curcumin at pH 5.5 compared to pH 7.0. This is due to the fact that liposomes are destabilized at a lower pH fostering the release of their content [37]. To evaluate the profile and mechanism of drug release *in vitro,* the amount of curcumin released was plotted against time and fitted in different release models such as zero-order, first order, Higuchi, Hixon–Crowell, and Korsmeyer–Peppas. The correlation coefficient (R2) was used to select the mathematical model that best fits the *in vitro* release profile and release mechanism of curcumin-loaded liposomes. The Higuchi mathematical model had the best fit as its *R*^2^ value was the highest for both acidic (pH 5.5-*R*^2^ 0.7917) and basic (pH 7.4-*R*^2^ 0.9679) media. This implies that the release mechanism for the curcumin was by dissolution as well as diffusion. The Higuchi mathematical model is applicable to study the release of poorly soluble drugs such as curcumin [38].

This model is based on the Fickian diffusion equation as seen in the following equation:

(3) $$\begin{array}{c} {Q=K}_{H}xt^{\left(1/2\right)}, \end{array}$$

where *Q* is the cumulative amount of drug at time *t*, KH is the Higuchi dissolution constant, and *t* is the time [38].

In this study, liposomes are formulated as suspensions; this means that dermal contact time will be limited due to its low viscosity. Hence, there is a need for liposomes to be incorporated into a triphasic viscous matrix system such as an organogel. Organogels are a three-dimensional gelled system in which apolar solvents are immobilized within their structure by organogelators (surfactant molecules) via capillary forces [39]. The ability to maintain a three-dimensional gelled system depends on the molecular structure and activity of the surfactant. There must be an equilibrium between the soluble and insoluble fractions of the surfactant in the solvent mix. It should be relatively insoluble enough to spontaneously assemble and form anisotropic structures. At the same time, it should possess a soluble fraction that will interact with the oil phase of the mixture [40]. Stable organogels can be achieved through chemical and physical interactions between the surfactant molecules, the oil, and the aqueous phase system. In this study, a ternary phase diagram reflects the three phases (Span 20: Tween 80, *Elaeis guineensis*, and water) as per their compositions at constant pressure and temperature [41]. The formation of organogels is dependent on the ratio of the three components. From the experimental results, it can be concluded that the proportion of the surfactants directly influences gelation, and this can be detected from the areas of gelation in the ternary phase diagrams. Organogels prepared using the ternary phase diagrams were evaluated for gel-to-sol temperature and stability.

The gel-to-sol transition temperature was determined by using the test-tube inversion method. The gel-to-sol transition temperature can be described as the temperature at which organogels transform from a solid-gel state to a more fluid state. This transition occurs as a result of the misalignment of the three-dimensional network structure formed by the interaction between the surfactant molecules and the apolar solvent. Once heat energy is transferred into the system, the entangled fibrous structures within the gel are disrupted, fostering a transition from gel to sol [42–44]. There is a direct relationship that is positive between the amount of kinetic energy needed to be introduced into an organogel system via heating and the degree of interaction between self-assembled structures formed by surfactant molecules. The transition temperature is a measure of the total amount of energy required to cause disruption of the gel network structure fostering sol-like flow in a previously gelled system [45]. Organogels formulated with cosurfactants at a ratio of 1:1 were generally more stable and had higher gel–sol temperatures. A1 was the most stable organogel from the results of the stability study. The formulation parameters for Formulation A1 were deployed in the formulation of the organogel used in F1, F2, and F3 and control.

The results of the organoleptic studies showed that the Formulations F1 and F2 had a characteristic golden yellow tint due to the presence of curcumin. F3 and control were translucent white. All formulations did not have any odor. All formulations had an emollient effect on application to the skin and were easily spreadable. The viscosities of organogels in Mini-Pascal were taken at body temperature at varying shear rate (10–100) rotations per minute. The rheological behavior of organogels was viscoelastic. This is because organogels showed both viscous and elastic properties. At lower shear rates, the organogel showed solid-like rheological properties; however, as the shear rate increased, there was a shift in plastic flow behavior. Stability studies showed that Formulations F1–F3 and control remained stable throughout storage [42].

Formulation F1 which contained both curcumin-loaded liposomes and chlorhexidine as active ingredients had the highest zones of inhibition for all three bacteria. This is due to the synergistic effect between curcumin and chlorhexidine [46]. Formulation F2 contains only curcumin-loaded liposomes and showed no activity against *E. coli* and *S. aureus*; however, there was some activity against *P. aeruginosa* (16.83 ± 0.62 mm). In a study by Ramesh et al, the minimum inhibitory concentration of curcumin against *P. aeruginosa* was 41.37 μg/mL [47]. The minimum inhibitory concentrations of *S. aureus* and *E. coli* were 46 and 885 μg/mL consecutively [48]. The concentration of curcumin in the liposome suspension is 42.195 μg/mL. This clarifies why Formulation F2 which contains curcumin showed activity against *P. aeruginosa* alone. Formulation F3 had chlorhexidine alone as the active ingredient, and it showed antibacterial activity against the three bacterial species though not as potent as Formulation F1. In a study by Sasidharan et al, the result of the antibacterial activity studies showed that curcumin potentiated the antibacterial action of cefaclor, cefpodoxime, and cefotaxime [49]. Similarly, in this study, it can be said that curcumin significantly enhanced the antibacterial effect of chlorhexidine from the antimicrobial results [50, 51]. The control formulation which did not contain curcumin nor chlorhexidine showed no antibacterial activity with no zone of inhibition.

The limitation of this study lies in the fact that *in vivo* studies were yet to be carried out due to the peculiarities of the research work (patients are infants). *In vivo* studies would require the application of this formulation on the infected navel of human infants. This qualifies as a Phase 1 clinical trial, which is currently out of the scope of this study. There are also ethical guidelines to consider in this regard. Another option would be to utilize primates for this study as rats do not have navels. This can be considered for future studies as an offshoot of this original research.

## 5. Conclusion

In this study, we successfully formulated stable curcumin-loaded liposomes in organogel. The release of curcumin from the liposomes followed a release pattern reflecting the Higuchi mathematical model. Liposomes were stable and about 7 μm in size with an encapsulation efficiency of about 97%. The *in vitro* release assay showed a sustained release pattern following the Higuchi kinetic model via Fickian diffusion. Organogel A1 was the most stable with a gel-to-sol temperature of 80°C and was used as a viscous vehicle for the curcumin-loaded liposomes and chlorhexidine. Formulation F1 had a pH of 6.5 and a spreadability of 63 mm^2^g^−1^ with plastic flow behavior on rheological assessment. Formulation F1 composed of curcumin-loaded liposomes and chlorhexidine in organogel was the most active in eliminating bacteria. The findings of this research proffer a novel effective and affordable treatment for omphalitis in infants, ultimately improving the overall health and well-being of infants in line with the third global sustainable development goal.

## Figures and Tables

**Table 1:** The different compositions of emulsifier mix, oil, and water for the formation of a stable triphasic gel system.

Organogel	Surfactant mix	% Composition		
		Surfactant	Water	Oil
A1	1:1	40	15	45
A2	1:1	35	20	45
A3	1:1	20	30	50
B1	1:2	40	15	45
B2	1:2	35	20	45
B3	1:2	20	30	50
C1	1:3	40	15	45
C2	1:3	35	20	45
C3	1:3	20	30	50

**Table 2:** The various compositions of ingredients for the preparation of loaded organogels and the control formulation.

Ingredients	F1	F2	F3	Control
Curcumin-loaded liposomes (mL)	10	10	—	—
Chlorhexidine (mL)	11.32	—	11.32	—
Span 20 (mL)	60	60	60	60
Tween 80 (mL)	60	60	60	60
Propylene glycol (mL)	0.025	0.025	0.025	0.025
Methylene glycol (ml)	0.3	0.3	0.3	0.3
*Elaeis guineensis* (mL)	135	135	135	135
Water (mL) to	300	300	300	300

**Table 3:** Gel-to-sol temperature of freshly prepared organogels, phase separation, and changes in color after 3 weeks of storage ([+] means phase separation, while [−] means no phase separation).

Formulation code	Gel-to-sol temperature °C		Phase separation		Appearance/color	
	Freshly prepared	After 3 days	25°C	4°C	25°C	4°C
Organogel A1	80	75	—	—	Cream to white	Cream to white
Organogel A2	60	40	—	—	Pale yellow	Cream to white
Organogel A3	55	45	—	—	Cream to white	Cream to white
Organogel B1	60	40	+	—	Cream to white	Cream to white
Organogel B2	55	25	+	+	Pale yellow	Pale yellow
Organogel B3	55	30	+	+	Pale yellow	Pale yellow
Organogel C1	55	30	+	—	Pale yellow	Cream to white
Organogel C2	55	30	+	+	Pale yellow	Pale yellow
Organogel C3	70	40	+	+	Cream to white	Pale yellow

**Table 4:** Results of the *in vitro* microbial study.

Organogels	Diameter of inhibition zone (mm)		
	*Escherichia coli*	*Pseudomonas aeruginosa*	*Staphylococcus aureus*
F1	22.00 ± 0.41	20.17 ± 0.24	24.33 ± 0.24
F2	—	16.83 ± 0.62	—
F3	20.17 ± 0.24	17.33 ± 0.24	20.17 ± 0.24
Control	—	—	—

*Note:* Results are expressed as mean ± S.D (*n* = 3).

⁣^∗^*p* < 0.05.

⁣^∗∗^*p* < 0.01.

⁣^∗∗∗^*p* < 0.001 with regard to significant differences.

## Data Availability

Data from this study can be made available on request.
